# Health care systems administrators perspectives on antimicrobial stewardship and infection prevention and control programs across three healthcare levels: a qualitative study

**DOI:** 10.1186/s13756-022-01196-7

**Published:** 2022-12-10

**Authors:** Isabel Naomi Aika, Ehijie Enato

**Affiliations:** Department of Clinical Pharmacy and Pharmacy Practice, Faculty of Pharmacy, University of Benin, Benin City, Nigeria

**Keywords:** Antimicrobial stewardship, Infection prevention and control, Programs

## Abstract

**Introduction:**

Antimicrobial stewardship (AMS) and infection prevention control (IPC) programs are proposed to address antimicrobial resistance. Nigeria’s 5-years action plan for these programs is expiring by 2022. The objective of this study was to examine the perspectives, barriers and facilitators of these programs among health care managers and the current state of these programs in the three levels of healthcare facilities in Benin City, Nigeria.

**Methods:**

Fourteen in-depth interviews were conducted among hospital manager across primary, secondary and tertiary healthcare facilities. The interviews were guided by semi-structured questionnaire. Responses were audio-taped and hand written. Data were analyzed by transcribing recorded tapes into major themes.

**Results:**

Most of the participants viewed inappropriate prescribing as a problem both in the country and their facilities. None of the institutions have a formal AMS program, but participants believed that the presence of drug and therapeutic committee is a foundation for such programme. Majority of the participants acknowledged barriers to AMS to include lack of management backing, interprofessional rivalry, and poor laboratories. Only the tertiary institution has a formal IPC program. Some challenges to IPC across the healthcare institutions are inadequate waste disposal, lack of personal protective equipment and behavioral change among healthcare providers.

**Conclusion:**

There is no AMS program across all facilities studied, although some forms of IPC activities are present in all healthcare institutions, only the tertiary facility has a formal IPC program. Effort should be made to strengthen AMS and IPC in the study facilities.

**Supplementary Information:**

The online version contains supplementary material available at 10.1186/s13756-022-01196-7.

## Background

Antimicrobial resistance is a serious public health problem requiring urgent health priority both at the national and international levels. The effects of antimicrobial resistance (AMR) extend beyond the individual as resistant microbes are transmitted among humans and between humans and the environment. This connection is clearly seen from hospital settings where patients who are admitted acquire infections from the environment. Inappropriate use of antimicrobial agents and poor infection control are directly linked to widespread AMR. In a systematic analysis, the estimated deaths attributable to AMR was highest in western sub-Saharan Africa at 27.3 deaths per 100,000 among adults aged 20.9–35.3 years [1]. In 2015, the World Health Organization (WHO) released a global action plan (GAP) on antimicrobial resistance. Antimicrobial stewardship (AMS) alongside infection prevention control (IPC) programmes are among the cornerstones of the GAP [2, 3]. Traditionally, these programs have been justified through measures of antimicrobial utilization with an associated reduction in the cost of antimicrobial therapy and changes in resistance rates and susceptibility patterns [4].

Nigeria along with many countries in low-middle-income-countries (LMICs) is yet to fully implement these programs especially in its health care facilities. In a situational analysis conducted in the country, widespread antimicrobial resistance pattern was observed among *Escherichia coli*, shigella, non-typhoidal salmonella, even among problematic antimicrobial resistant organisms such as carbapenem-resistant enteriobecteriacea, vancomycin-resistant enterococci and extended-spectrum beta-lactamase-producing gram-negative rods to salvage antimicrobials at an alarming rate [5]. Against this backdrop, and in tune with the global call for action plan on tackling antimicrobial resistance, the Federal Government of Nigeria through the Ministry of Health established the country’s antimicrobial resistance coordinating body at the Nigerian Centre for Disease Control (NCDC) with key stakeholders to provide support and guidance for AMR control activities in the country. The National Action Plan (NAP) as road map to curbing AMR was developed from weaknesses identified in the situation analysis report. A 5-focus strategy is adopted in the plan to address the problem of AMR within a 5-year period (2017–2022) through intensifying establishment of a national infection prevention and control (IPC) program and antimicrobial stewardship programs and strengthening these programs across all healthcare levels [6].

It is observed that presence of policies in LMICs may not guarantee their implementation due to lack of strong leadership commitment [7]. This behavior can trickle down at the healthcare facility level which in addition to other barriers can impede to implementation of AMS and IPC programs across the country. It is imperative that individual healthcare facility takes measures to implement AMS/IPC programs until the expected national input reinforces their effort. Understanding the perspectives of hospital and health managers to AMS is vital towards the establishment of robust and sustainable antimicrobial stewardship programs and the engagement of management in addressing potential blockades to change [8]. This study sought to describe the perspectives, barriers and facilitators to AMS and IPC programs among health managers and the current state of these programs in the three levels of healthcare facilities in Benin City, Nigeria.

## Methods

### Study design/setting

This was a cross-sectional qualitative study conducted between September 2020 and July 2021. The study was conducted in Benin City, Edo State, located in Southern Nigeria. It has a population of about 5 million inhabitants. There are 3 tertiary, 38 secondary and 576 primary public healthcare facilities in the 18 local government areas of the state [9]. The tertiary facilities are run by the federal government, while the state hospitals are managed by the state government through the Hospital Management Board (HMB). The primary health centers (PHC) are under the management of local government but currently run by the state government. The study was carried out among healthcare managers across the three levels of care.

### Data collection

A semi-structured interviewer’s questionnaire was used as data collection tool (Additional file 1: S1-Questionnaire for Qualitative IPC study; and Additional file 2: S2-Questionnaire for Qualitative AMS study). The questionnaire was in two sections. Section one provided details of participants demographics, while section two was a 15-item questions developed from extensive literature review on AMS and IPC programs. Face -to- face interviews were conducted using the questionnaire, the interviews were audio-taped using a phone as the recording device, it was also accompanied by hand written notes of the responses. Participants were allowed to express their thoughts on issues relating to antimicrobial resistance, antimicrobial stewardship, antibiotics use, antibiotics prescription pattern and infection prevention and control practices and in some cases, follow up questions were asked to ensure sufficient clarity on participants responses on these issues.

### Recruitment of participants

A total of fourteen participants were interviewed. Participants were purposively selected**.** Respondents were coded as Participants T (T1–T5) for interviews from tertiary healthcare facility, S (S1–S5) and P (P1–P4) from secondary and primary facilities respectively. Participants interviewed on AMS in the tertiary and secondary facilities were drawn from members of the Drug and Therapeutics Committee (DTC). T1 is a medical doctor, T2 is the head of pharmacy department, T3 is a medical microbiologist and chairman of the proposed AMS arm of the DTC, S1 is the head of surgical department and a medical doctor, S2 is the head of pharmacy unit, while S3 is a medical doctor and head of medical department. Participants recruited for IPC interview at the secondary level were drawn from the state Ministry of Health and HMB and they comprised of the Director of Nursing Services (S5) working with the focal person for IPC activities and reporting in the state (S4); for the tertiary institution, a medical doctor who heads the IPC unit and serves as chairman of the hospital’s IPC committee (T4) and the chief nursing officer for IPC (T5) were recruited; at the primary healthcare facilities, four coordinators who are medical doctors drawn from four local government areas-Ovia North East (P1), Ikpoba-Okha (P2), Oredo (P3) and Egor (P4) participated. All participants were approached face-to-face to arrange for a convenient day and time for the interview. The average time of the interviews was 20–45 min.

### Data analysis

The audio-taped interviews were reviewed and transcribed into notes based on the emerging themes. The data were interpreted within a deductive thematic analysis framework. The data were analyzed using a directed content analysis approach. Specifically, the analytical process involved independently reviewing all transcript line by line to identify text, statement or comments that fall under some predetermined themes and categories. Subsequently quotes reflecting each subtheme were categorized, and corresponding descriptions were developed, leading to reorganizing, renaming and elimination of some themes and subthemes. This procedure was then repeated for themes and subthemes requiring further analysis. Data analyses were iterative, whereby themes, subthemes and sub-categories were added to reflect variations in the data (Additional file 3: D1-AMS Qualitive Transcription; and Additional file 4: D2—IPC Qualitative Transcription).

## Results

### Theme 1: current state of antimicrobial use

Regarding the current use of antimicrobial agents, all respondents said that irrational use of antibiotics is a major problem in Nigeria. S2 noted “there’s no guided use, anybody just says this is what they want. As a pharmacist, we try to restrict use, but the prescribers will say by the time patients come to them, they have used several antibiotics thus leaving them with no choice but to use higher class or new generations of antibiotics. The medical representative’s influence cannot be neglected. They give data of new antibiotics or fixed-dose combinations to prescribers, those in turn end up yielding and prescribing these drugs”.

The most common antibiotic use problem identified is wrong selection of antibiotic followed by overprescribing. The issue of inappropriate prescribing may tend to be higher in PHC settings of which majority do not have laboratories and some have nurses or community health extension workers as managers. Many of these facilities admitted to having access to antibiotics that should ideally be restricted for use in primary care such as floroquinolones and cephalosporins, and it seems some of the personnel do not understand antibiotic use restriction (P1, P2)*.*

### Theme 2: laboratory and antimicrobial resistance surveillance report

The secondary and tertiary healthcare facilities have laboratories. In the primary healthcare centres, only one from the four local government areas sampled has a laboratory in the local government headquarter where culture and sensitivity tests can be done. The others refer patients to other laboratories (either in the state hospital or privately owned (P1, P4). The tertiary institution alone has regular surveillance report only on healthcare associated infection (HCAIs), this is part of the activities carried out from the stand-alone infection control unit that gives updated reports. Respondents linked antimicrobial resistance to patients who have either self-medicated or have been given antibiotics in drug outlets like patent medicine dealers or pharmacies and to poor infection control in hospitals. The pattern of resistance in HCAIs shows coagulate negative, Staphylococcus aureus, Pseudomona aereuginosa, Klebsiella specie, Providential species, Enterobacter, and Citrobacter, alkaliginase. The highest resistance being coagulase negative Staph aureus, and the lowest is Alkaliginase and Citrobacter (T4)*.*

Regarding resistance to antimicrobial agents, S1 pointed that “from lab results, the sensitivity pattern can be very discouraging. Common ones like Penicillins (amoxyl) Cephalosporins (Ceftriaxone) etc. are no more effective, some organisms are only sensitive to floroquinolones and imipenem which are not cheap, it therefore becomes difficult to start requesting for drug like imipenem to treat common urinary tract infection”.

### Theme 3: presence and functionality of formal antimicrobial stewardship practice

The idea of AMS in practice setting was welcomed by all responders in the tertiary and secondary facilities as a means to restrict antibiotic use, unfortunately none of the facilities have a formal AMS program. Most participants identified the role of government policy as key to institutionalizing AMS, in addition to providing national guideline on antimicrobial use which is currently lacking. T2 mentioned “Government involvement if it is committed will have positive impact, in addition to policy makers. I am not aware of antimicrobial guideline, what I am aware of is guideline for use of drugs in hospitals i.e. standard treatment guideline. I can’t say it’s being in use”.

At the primary healthcare level, the concept of antimicrobial stewardship was vague to most of the participants, thus they were briefly enlightened. P4 said, “I don’t really understand what it means. But if it has to do with effective use of antibiotics then it is a program that should be encouraged and taken seriously”.

### Theme 4: likely barriers to AMS

Participants identified barriers to antimicrobial stewardship in their facilities. Major barriers are lack of management commitment and interprofessional rivalry which cuts across both secondary and tertiary facilities. Other barriers are shortage of professionals and poorly equipped laboratories (Table 1).

**Table 1:** Barriers to AMS

Barriers	Response
Management backing/commitment	T3: “ AMS is difficult because we are going to experience friction e.g. autonomy in auditing prescriptions, without management backing it won’t work” T2: “there is no commitment from the leadership of the hospital. In Nigeria, we have this general attitude of not implementing policies”
Interprofessional rivalry	S2: “likely barrier is our professional rivalry, it’s for everybody to realize that we are in this workplace for the benefit of patients” T3: “one of the things with bringing guideline for hospital use is if the hospital has an antibiogram. To do this effectively, we need very good working relationship with pharmacist, doctors, clinical microbiologists etc. in most institutions in Nigeria, there is this rivalry that may make it difficult to work together seamlessly”
Shortage of healthcare professionals	S3: “In the state, generally, there’s a dearth in manpower. Imagine no qualified staff to run those specialized laboratories” T3: “AMS is an intensive program, we are few with other professional duties, so there will be need for dedicated staff”
Poor laboratory services	S3: “in the institution, reporting is a problem, poor laboratory services like we talked about. Antibiotic resistance involves testing”
Lack of training for healthcare professionals	S1: “In my institution, adequate education of health professionals and update on current use of antimicrobial guidelines is a big challenge”

### Theme 5: facilitators to instituting AMS

Table 2 shows participants perspectives on what their facilities have on ground that can facilitate the implementation of AMS. Most of them pointed to the presence of DTC which can be leveraged on.

**Table 2:** Possible facilitators of AMS

Facilitators	Response
The presence of an already functional Drug and Therapeutics Committee (DTC)	T1: we already have a crop of professionals who are interested in this program, the necessary professionals are present in this hospital T2: the DTC has been inaugurated and can be built on S2: DTC, currently due to staff strength depletion, the committee is not meeting up to its responsibility. It’s a new committee. The pandemic made transfer of staff to the isolation center for Covid, that has affected its running S3: the DTC which is functional. we have already set up an AMS group which if not for Covid would have kick-started. Our present leadership is kind of interested in making it happen T2: DTC in the hospital, we have taken the initiative of grooming a pharmacist to go for training and inform the management through the DTC
Information technology unit	T3: The information technology unit in the hospital can be leveraged on. For example, the antibiogram I talked about can be generated from the infrastructure already on ground

### Infection prevention and control (IPC) programme

#### Theme 1: thoughts about IPC

Some participants reported that the recent pandemic highlighted the importance of IPC in healthcare systems. Only the tertiary hospital reported having a formal IPC team with a reporting channel, funding and guideline. In the secondary and primary facilities there is no formal IPC, but IPC activities are carried out by the IPC pillar an offshoot from COVID pandemic charged with the responsibility to oversee the IPC activities in all state hospitals (T5, S4). One of the participants S5 from the state Ministry of Health noted “I don’t think we have a formal IPC as described, but part of what Edo State IPC pillar is doing is to inform the various hospital and the public on prevention of nosocomial infection which is key to preventing spread of infection”.

All respondents from PHC mentioned that they are under the State’s IPC program and they receive equipment for their IPC activities from the State’s team. Most respondents revealed that the head of the PHC coordinates IPC activities in the facility and reports to the PHC coordinator of the local government who in turn reports to the State coordinator of IPC.

#### Theme 2: role of training in IPC activities

All facilities engage in training and retraining on a regular basis, some respondents noted that they have attended at least a training on IPC in the past 1 year (P3, P4). T4 mentioned “last year alone, we had nothing less than 8 trainings, educating healthcare workers on hand hygiene and waste management, because we know that the hands of these healthcare workers are the most important vehicle for transmitting infections.”

S4 added “that’s the bedrock of IPC. Training helps to reinforce knowledge already acquired and also add new knowledge”.

#### Theme 3: provision of adequate personal protective equipment (PPEs)

The tertiary institution has a logistic unit that supplies all PPEs. The COVID-Pillar which is an arm of the state’s COVID committee provides PPEs to all state hospitals and PHCs. However, some respondents reported that provision of such materials are not sufficient and that they had to buy some IPC materials with their personal money (P2, P1, P3). T4 commented on some innovations the unit made recently to cope with inadequate PPEs, “we have a logistic unit that ensures we have all PPEs. In fact, we had an indigenous production of some PPEs especially during the hit of COVID as there was tendency of worldwide shortage of PPEs, we started making things, cloth face masks for our admin personnel, long sleeve covers, aprons, face shield. We also made UV light equipment to disinfect some of our PPEs like face shields and eyewear”.

#### Theme-4: adequate waste disposal, clean and safe environment

All responders said they had a system of waste disposal, but only the tertiary facility have good waste segregation into infectious, non-infectious, highly infectious and sharps (T4, T5) At the secondary level one participant (S5) explained the inadequacy in the waste disposal system “I think we still need training on segregation where the waste is generated, we just dump everything together which can be risky for those disposing it eventually, that is the scavengers, then good sterilization is needed especially in rural areas where they boil their instruments, that will help”.

Most of the respondents in PHC facilities stated that they take wastes that require incineration to University of Benin Teaching Hospital or the World Health Organization waste disposal facility (P1, P2).

Some participants reported that things are put in place to ensure safe and clean environment in their facilities. Cleaners are trained on proper cleaning practice and waste management (S5, P4, T5). Participant T4 detailed “Our environment is regularly cleaned. Now and during the hit of COVID, we use 0.5% sodium hypochloride solution to clean the floor, the walls and high touch areas such as door knobs and switches even the beddings and mattresses, and everything with same solution. We clean the wards and clinics at least twice daily.”

#### Theme 5: hand hygiene practice in IPC

Compliance with hand hygiene practice was reported to be generally good at the all facilities, although many participants agreed that there is a decrease in hand washing compared to a year ago when we were in the heat of the COVID-19 pandemic which heightened the level of awareness to strictly adhering to hand hygiene measures among healthcare providers. At the secondary facility, S2 gave a positive response and added areas of improvement “People are strict about it. Each unit has wash hand basin and water. Management has provided liquid soap too. But we can do better in a modern way, instead of bucket we should have running taps in work stations and toilets. Nowadays, you don’t even touch tap heads, they use sensors, same thing goes for soap, this helps control/prevent infection”.

#### Theme 6: challenges, strength and sustainability of IPC activities

Limited supply of waste disposal materials and PPEs were cited in all facilities as major challenges. In the primary and secondary facilities, lack of transportation and shortage of staff hinder waste disposal and other IPC activities. In the tertiary institution, behavioural change or lack of compliance especially by new healthcare workers is another limitation observed, it was noted that other healthcare providers still need to imbibe the culture of IPC activities notably hand hygiene instead of doing it out of fear of getting infected (T5, S4, P1, P3).

Regarding the strengths of IPC activities, the tertiary institution highlighted management commitment for allowing the IPC team to function independently. Although no formal IPC in both secondary and primary institutions, most respondents praised the government for the provision of PPEs compared to before even if they are sometimes not sufficient and training of healthcare workers as the strength of IPC, they were optimistic that likely in the future the ideal IPC program will be practiced (T4, S4, P1, P3, P4).

Regarding sustainability of IPC activities, T4 said “as long as the IPC committee is sustained in the facility which is over 20 years now, plus an enabling environment for the IPC committee to work, it will go far and be sustained”.

S5 said “on a scale of 1–10, I will say 5. You know as government changes, things can change”.

## Discussion

This study sought to examine antimicrobial stewardship and infection prevention control activities across the three levels of healthcare in Benin City, Nigeria. Irrational use of antimicrobials was noted in all healthcare settings, there are no restrictions of antimicrobial use even in primary healthcare centers where antibiotics in the WHO watch category are often prescribed to patients [10]. It has been estimated that about 30–50% of antibiotic consumption in hospitals in LMICs is inappropriate. Factors contributing to this include lack of regulation and misuse of antibiotics for treatment of viruses causing upper respiratory tract infections and acute bronchitis in the community [11]. In rural and under-resourced settings of these countries, where access to qualified healthcare workers is severely constrained, universal health coverage has been erroneously equated with the availability of antimicrobials [12].

### Antimicrobial stewardship program (AMS)

There’s is no formal antimicrobial stewardship in all facilities sampled, the presence of DTC was perceived as a facilitator to starting AMS program. Such finding was reported in Kenya among healthcare professions [8]. In many low-resource settings, adaptations have been made where it is difficult to have a stand-alone AMS team, similar committee as the IPC or in this case DTC and AMS committees may be merged into one. This method was adopted in Barbados where the AMS was linked with existing IPC programme during an outbreak, this subsequently led to creating an AMS stand-alone committee [10]. In some small facilities, an AMS champion can be identified instead who can either be a pharmacist or a nurse, this often eliminates the issue of staff shortage. This method has been successfully adopted and sustained in many hospitals in South Africa [13, 14]. This option will offer an opportunity for PHCs to control simple infections, thus reducing the reliance on over-the-counter medications and self-medication. Participants suggested that government policy and implementation of AMS in all health facilities will prove to be instrumental in formalizing AMS. Among the barriers identified in this study are three from the core elements for AMS programmes toolkit by the World Health Organization, these are; lack of management commitment, lack of training of healthcare professionals, and poor laboratory services for surveillance [10]. The need for hospital managers to be committed and accountable with respect to AMS cannot be overemphasized. Even with the existence of government policy, they play a significant role in determining the value of an AMS to the institution otherwise such programs have a tendency to fail. Besides, recalcitrant prescribers may thwart attempts to improve antimicrobial use without fear of sanction [4, 15]. Absence and poorly equipped laboratory impede AMS activities. Antibiograms are usually developed based on laboratory reports and are regularly updated based on a review and analysis of facility antibiotic use and antibiotic resistance. The antibiogram may help to inform updates on clinical guidelines [16, 17]. In a report by the Nigerian Center for Disease Control, only about 6% of public health facilities in Nigeria have a laboratory, and two-thirds of these laboratories do not have adequate qualified personnel to handle the assigned diagnostic tasks [5]. Healthcare professionals who are AMS champions require adequate initial and ongoing training on AMR, antimicrobial prescription behaviour and use of standard treatment guidelines. Lack of antimicrobial use guideline is contributory to inappropriate prescribing in hospitals, as some respondents noted. Healthcare facilities should have available, up-to-date recommendations for infection management based on international/national evidence-based guidelines and local/national susceptibility patterns (where possible), to assist with antibiotic selection for common clinical conditions (indication, agent, dose, route, interval, duration) [18]. Enhancing the availability of guidelines for frequently encountered infections and clarifying key guideline recommendations such as treatment duration were identified as effective AMS interventions in a survey of hospital staff across 58 LMICs including primary healthcare centers [7].

Inter-professional rivalry is a foreseen challenge to AMS that should not be overlooked. Teamwork in the Nigerian healthcare system is marked by interprofessional disputes that are very intense. Some surveys in the country have suggested the overwhelming recognition of interprofessional conflicts by health professionals, with perceived differential treatment between the professions, the assertion of role boundaries, and communication barriers as predominant causes. In terms of its impact on health workers, one survey in the North-Eastern region found interprofessional conflicts to be associated with diminished motivation [19, 20]. Until the drivers of interprofessional rivalry are addressed and all members of the healthcare team share common goals, AMS program will suffer some setback when it eventually kicks off. An important solution to this conflict is to apply behavioural change communication through repeated training of health professionals. Efforts should focus on the key message that all professional groups are important in the delivery of healthcare services and to learn collaborative patient care, interprofessional respect and clarity of roles. Secondly, equal opportunities to leadership positions should be provided to all qualified professionals and remuneration gap must be closed or reduced. This second approach may take longer to come to reality because it will involve deliberations among professional groups with government to change the current state. Hence, the first approach in within reach of individual health professionals and institutions [19, 21].

### Infection prevention and control programs

Infection prevention and control activities are present in all healthcare facilities, clearly the recent COVID-19 pandemic brought the need for such programmes to limelight. Only the tertiary institution has a formal IPC unit. In the secondary facilities and by extension the primary care centers that are under the management of the state government, IPC activities are channeled through the COVID-pillar an arm that is dedicated to reducing infection and spread of coronavirus.

Standard precautions are the core component of IPC, it includes appropriate use of personal protective equipment, environmental cleaning/disinfection, medical waste disposal, and hand hygiene practices [22]. When implemented correctly by health workers, these precautions keep the worker protected from infection and prevent infection from spreading among patients [23]. Participants in this study disclosed that to some extent they comply with the use and provisions of these components. The tertiary institution seems to have better efficiency in IPC programme clearly due to the dedicated unit, this underscores the benefit of having such stand-alone unit in healthcare facilities. Management support and dedicated team with guideline have been reported to be the strength of successful IPC activities [22, 24].

Some drawbacks to IPC activities across all healthcare facilities as noted by participants are shortage of PPEs, inadequate waste disposal measures and behavioral change by healthcare providers. In the face of the ongoing pandemic, inadequate PPEs turned into a global issue as the need for such became heightened. A study in Nigeria also described such shortages especially among frontline workers, with such shortages, there’s tendency to share these items among workers, reuse or even attend to patients bare, with the attendant risk of transmitting infections [24]. In the tertiary institution, one way the IPC unit accommodated for the shortage was to make indigenous PPEs, this action can be replicated in other healthcare institutions in the country.

Proper waste segregation denotes separating wastes in coded bins as non-infectious, infectious, highly infectious and sharps. Inappropriate waste segregation and disposal means that all waste is treated the same, leading to improper segregation and making the total waste infectious. This causes a huge problem not just for the waste handlers but for the rest of the population. Many healthcare facilities in Nigeria do not have resources such as incinerators and consumables to properly treat healthcare wastes (sometimes hazardous and infectious) before disposal [25]. As seen from these interviews, the PHCs do not have such facilities, they have to travel far to the closest incinerators, the same thing goes for the secondary healthcare facilities, if those in the city center where the study was conducted lamented over the issue of waste segregation, disposal and poor transportation facilities, those in farther local government areas and rural communities obviously bear more burden.

Proper Hand hygiene is a significant component of IPC, this simple evidence-based practice has been shown to have great impact on reducing hospital acquired infection and antimicrobial resistance [26]. This study shows that there’s good compliance to hand hygiene, like some participants noted, “it is the good side of COVID-19”. Although this practice is still valued by the public and healthcare professionals in particular, some participants described a drop in the intensity of the practice compared to the hit of the pandemic. Behavioural change and poor hand washing infrastructure were noted for this observation. Some facilities improvised during the hit of the pandemic with mobile plastic buckets located in strategic places, but now few of these buckets are available. The burden of moving these buckets to get water, bringing them to their stations and discarding the used water can be discouraging, difficult to sustain and time consuming. These challenges were noted across all healthcare facilities studied, but more in the secondary and primary care areas. Similar challenges were reported in a study among 8 countries in Africa including Nigeria, a number of sites lacked functional water points in patient care area [27]. It is challenging to prevent the spread of infections within and outside healthcare settings if the basic infrastructure to improve hygiene and sanitation is lacking.

All participants remarked positively as to the role of training in IPC. Training and retraining remind healthcare professionals on strict adherence to IPC measures and are key in forestalling the impact of unforeseen epidemics [28]. While the importance of training cannot be overemphasized, it alone is not enough to change one’s culture. In a study conducted in Africa, participants noted the need for behavioural change interventions (another drawback to IPC compliance), monitoring and follow-up in addition to in-service training. Hand hygiene and waste segregation are widely recognized as practices that need continuous reinforcement just like other behaviour that require change in medical settings [29].

The strength of this study lies in the fact that responses where from key informants, for AMS those involved in decision making on drug-related use in the healthcare facilities, and for IPC, those who are directly involved in IPC activities either in their facilities alone or by extension in the state. This gives a near representation of the state of these activities in Edo State. A limitation to this study is that it may not be a representation of what happens in the country at large.

## Conclusions

This study has showed non-existence of AMS practice across healthcare levels in the study area, and likely barriers. Healthcare professionals expressed willingness to adopt AMS practice with the DTC which is functional to be a starting point. The various healthcare facilities are actively involved in IPC programs, the tertiary facility have more robust programme because of the dedicated IPC unit compared to other institutions. The study also identified some lapses and challenges in IPC across all facilities; inadequate waste disposal facilities and behavioural change are some drawbacks. Considering the fact that the 5-year plan for implementing these programs will soon elapse, legislative action, funding, and public policy strategies are needed, hence government and hospital administrators/managers need to work more closely to achieve the aims of the country’s action plan to curb antimicrobial resistance. The need for strengthening PHC facilities (particularly manpower and diagnostic infrastructure) as a means of reducing health inequalities, including controlling simple infections, hence reducing the reliance on over-the -counter medications is revealed in this study.

## Supplementary Information

**Additional file1**. **S1**: Questionnaire for Qualitative IPC study.

**Additional file2**. **S2**: Questionnaire for Qualitative AMS study.

**Additional file3**. **D1**: AMS Qualitative Transcription.

**Additional file4**. **D2**: IPC Qualitative Transcription.

## Data Availability

S1: Questionnaire for Qualitative IPC study. S2: Questionnaire for Qualitative AMS study. D1: AMS Qualitative Transcription. D2: IPC Qualitative Transcription.
