# Supplementary material for: Health care systems administrators perspectives on antimicrobial stewardship and infection prevention and control programs across three healthcare levels: a qualitative study
Source: Antimicrob Resist Infect Control. 2022 Dec 10;11:157. doi: 10.1186/s13756-022-01196-7 (PMC9739345; doi:10.1186/s13756-022-01196-7)
Supplement: Supplementary file 1 — Additional file1. S1: Questionnaire for Qualitative IPC study. [file 13756_2022_1196_MOESM1_ESM.docx]

**QUESTIONNIARE… QUALITATIVE STUDY (Key Informant Interview Guide on Infection Prevention and Control Practice [IPC] )**

| **Socio-demographic Questions** | | **Response** |
| --- | --- | --- |
| 1 | Age in years­­ | __________ years |
| 2 | Gender | 🞏 Male 🞏 Female |
| 3 | Level of healthcare institution | 🞏 Primary 🞏 Secondary 🞏 Tertiary |
| 4 | Position in this hospital |  |
| 5 | How long have you worked in your current specialty or profession? | ______________years |

**Questions**

- 1. Please explain your clinical role and how it relates to your hospital’s overall infection prevention strategy?
  2. Do you think inappropriate use of antibiotics is a problem in Nigeria and in your institution? How do you describe the pattern of this problem over time?
  3. How frequently do you encounter hospital acquired infection and resistance within your clinical practice? Which types of pathogens/infections do you think have more resistance profile?
  4. Do you have a regular surveillance report of resistance and susceptibility pattern shared among healthcare workers?
  5. What are your thoughts about infection prevention and control practice (IPCP) in Nigeria healthcare institutions? Can you narrow this to your institution?

**Infection Prevention and Control Program**

- 1. Does your hospital have a formal IPC program?

If yes, can you say in an ideal sense that it is functional? Can you describe the structure and composition in terms of (i) formal policy(ii) Dedicated Team (iii) local guideline (iv) Tracking and feedback system (v) Documentation of activities

- 1. What is the role of training in your activities and how often is it done?
  2. What can you say about the following in IPC program in your institution;

(b) Adequate personal protective equipment (b) Adequate waste disposal (c) Ensuring clean and safe environment

9. What are your thoughts about your preparedness in the event of an epidemic or case identification since we are in an era where such eventualities are becoming more frequent

10. What are the challenges you encounter to implement programs in your institution? And what do you think can be done to improve your activities

11. What do you think are the strengths of IPC practice in your institution?

12. How do you see the sustainability of your activities in years to come

13. Comparing hand hygiene now and in the past what do you have to say

14. What are you doing to encourage interdisciplinary collaboration

15. How is your team embracing the use of technology to advance IPC activities
