# Supplementary material for: Health care systems administrators perspectives on antimicrobial stewardship and infection prevention and control programs across three healthcare levels: a qualitative study
Source: Antimicrob Resist Infect Control. 2022 Dec 10;11:157. doi: 10.1186/s13756-022-01196-7 (PMC9739345; doi:10.1186/s13756-022-01196-7)
Supplement: Supplementary file 2 — Additional file2. S2: Questionnaire for Qualitative AMS study. [file 13756_2022_1196_MOESM2_ESM.docx]

**QUESTIONNIARE… QUALITATIVE STUDY (Key Informant Interview Guide on Antimicrobial Stewardship Program)**

| **Socio-demographic Questions** | | **Response** |
| --- | --- | --- |
| 1 | Age in years­­ | __________ years |
| 2 | Gender | 🞏 Male 🞏 Female |
| 3 | Level of healthcare institution | 🞏 Primary 🞏 Secondary 🞏 Tertiary |
| 4 | Position in this hospital |  |
| 5 | How long have you worked in your current specialty or profession? | ______________years |

- 1. Do you think inappropriate use of antibiotics is a problem in Nigeria and in this institution?
  2. Which problems are more prevalent or common in this setting? How do you describe the pattern of this problem over time?

**Antimicrobial Resistance**

- 1. What can you say about antimicrobial resistance (in practice)?

Do you usually have a regular surveillance reports of resistance and susceptibility pattern

Shared among healthcare workers?

What factors do you think are contributing to increasing emergence and spread of antimicrobial resistance? Please explain on how commonly each of them exist in your hospital?

**Antimicrobial Stewardship Program**

- 1. What are your thoughts about Antimicrobial stewardship programs in Nigeria?

What are your thoughts about the government’s involvement on AMS in the country with regards to the national action plan for antimicrobial resistance? How will you describe the current use of antimicrobials in your facility in terms of appropriateness?

- 1. Does your hospital have a formal AMS program?

If yes, can you say in an ideal sense that it is functional? can you describe the structure and composition in terms of (i) formal policy(ii) Dedicated Team (iii) local antibiotic treatment guideline (iv) Tracking and reporting system (v) dedicated funding (vi) monitoring and evaluation.

1. Does your institution have antimicrobial consumption data at both patient level and facility level?
2. What do you think are the barriers to AMS in your institution and the country at large?
3. What can you say about how to mitigate these barriers?
4. What do you think are the facilitators of AMS programs in your institution that you can build on?
5. Do you think your institution has the capacity to fully implement, be committed to and sustain AMS program?
6. A key aspect of infection control is hand hygiene, what can you say about this practice among healthcare professionals in your institution?

Alcohol based handrub/water and soap always available at all hand washing basins? (ii) Enough hand washing facilities in your institution (iii) Routine education/ training of staffs

**That’s the end of the interview. Thank you for your time!!!**
