# Supplementary material for: Health care systems administrators perspectives on antimicrobial stewardship and infection prevention and control programs across three healthcare levels: a qualitative study
Source: Antimicrob Resist Infect Control. 2022 Dec 10;11:157. doi: 10.1186/s13756-022-01196-7 (PMC9739345; doi:10.1186/s13756-022-01196-7)
Supplement: Supplementary file 3 — Additional file3. D1: AMS Qualitative Transcription. [file 13756_2022_1196_MOESM3_ESM.docx]

**Antimicrobial Stewardship Program (AMS)** **Qualitative Interview Transcription**

- 1. **Do you think inappropriate use of antibiotics is a problem in Nigeria and in this institution?**

S1; we try as much as possible to use antibiotics rationally, we use antibiotics more with sensitivity test results. Global practice in hospitals in Nigeria, however, we know use of over-the-counter (OTC) drugs is prevalent in our country which is a big issue.

S2: I will say there’s no guided use, anybody just becomes and says this is what they want. As a pharmacist, we try to restrict use, but the prescribers will say by the time patients come to them, they have used several antibiotics thus leaving them with no choice but to use higher class or generation of antibiotics. The medical representative’s influence cannot be neglected. They give data of new antibiotics or fixed-dose combinations to prescribers, those in turn end up yielding and prescribing these drugs.

S3: I will speak more of my institution. i will say from the current trend, it is rational within the ambient of practice.

T1: it is a bit chaotic in that anybody can have access to antibiotics no matter the class, even those restricted. In the hospital here, I want to say doctors are more prudent in using them.

T2: in Nigeria and my institution, it is still not rational, because we still see conditions where some doctors prescribe antibiotics for viral infections. In terms of dosing, some doctors prescribe under or overdoses, it boils down to the fact that drugs are many a times prescribed empirically.

T3: to a large extent, it is guided by lab results, although empirical approach is used before results come out. Antibiotics are used based on the discretion of the managing physician, because we do not yet have our own localized institutional guideline for various infections, so it is unit-based. For example, in the respiratory unit-based on likely organism suspected for causing RTI, patients are given antibiotics. The stage where we are as an institution is more like an exploratory phase, trying to see what the baseline is so that when we introduce definite policies and guidelines, we can compare what individual doctors are doing and as compare with guidelines.

P1: Antibiotics are obviously misused in our country as there is free access.

P2: There is inappropriate use of antimicrobials in many facilities. I say so because majority of patients that use antibiotics do not usually do culture and sensitivity test, a few that do, they have already started antibiotic treatment before result is out.

P3: Antibiotics are generally overprescribed. In some centers headed by nurses or health care workers, their knowledge and use of these drugs are limited, we find them giving patients antibiotics for conditions they are not indicated.

P4: Antibiotic use is not regulated at all and it is not good for our health system.

**2. Which antibiotic use problems are more prevalent or common in this setting? How do you describe the pattern of this problem over time**?

T2: As a clinical microbiologist, I get consultations on advice for antibiotics, sometimes we do see inappropriate use of antibiotics. (b) under-dosing is not of a problem. Here it is selection of antimicrobials and using antimicrobials where there is no evidence for it. E.g in the paediatric age group, when patients come down with a throat infections, which often time is of viral origin, we see a lot of antibiotics used (instead) even when samples sent for culture do not show evidence of bacterial infection. That feeling of not comfortable if antibiotics are not given also plays a part. It is a normal feeling especially when patients expect to receive antibiotics when they are not needed. Another thing is using broad-spectrum antibiotics when narrow-spectrum ones can suffice. World Health Organisation has classified antibiotics as access, watch and reserve. There’s this tendency to use reserve antibiotics first which are normally used for resistant microorganisms. (c) trend, I cant really say for sure, we are to conduct survey of antibiotic use to have a point-prevalent data, then periodically we can do another survey to compare.

T1: I can say it is increasing. Like the instance of viral infections I mentioned that they use antibiotics for. When doctors see infection, they don’t evaluate the cause whether viral, helminthic, protozoa etc. antibiotics is the quick solution.

S3: the role of laboratory is important. Some doctors usually request for lab investigation and start empirical treatment before results come out. But the turn out time is usually an issue. There should be synergy amongst healthcare workers.

S3: Antimicrobial misuse is a problem in my institution and Nigeria (b) over prescribing and under dosing. Frequency/duration of therapy. (c) maybe its stable. It’s almost a norm.

S2: There is misuse of antibiotics not prescribed and not taking prescribed dose. But in the hospital here, I believe they are prescribed when needed. Since they do sensitivity test, although they sometimes do blind (empirical ) treatment, but that changes when results come out. Still some doctors do not order for sensitivity tests before commencement of treatment which not only leads to irrational prescribing but increases cost of treatment for patients.

S1: in my institution, I will be lying by saying no inapproproite antibiotic use issue outrightly, because few people prescribe wrong antibiotics and give a blanket cephalosporins and flagyl kind of antibiotics prescription, however, majority tend to like I said prescribe based on common microbial flora that are known for certain diseases.

**3. What can you say about antimicrobial resistance (in practice)?**

**(B) Do you usually have a regular surveillance reports of resistance and susceptibility pattern shared among healthcare workers?**

S2: the resistance I normally see are for patients already on antibiotics prior to meeting me, that doesn’t happen often, but I would say, maybe once or twice, patients whose culture tend to be resistant against all antibiotics used in the hospital. (B) as much as we would like to have that, but since I came here no, it hasn’t happened.

T3: I don’t know if it is documented, but there are, out-patient will be more guilty than in-patients, because in in-patient, they switch once sensitivity results are available. (B) Not really

S3: very rare, because it is not within our job description to know. Those in the lab may have such information (B) Not in the pharmacy, only recently the DTC set up an AMS group, we have not really started working, when we do, we might have this data.

T1: I can’t say categorically that there are cases of resistance, because there is no monitoring.

S1: yes we do, even if we do not have statistics or records, we’ve seen cases where patients have been seen in chemists, private hospitals and other outlets. If we take drug history, we discover that they have used antibiotics before, so one is tempted to step higher since the basisc antibiotics are not effective. (b) yes, from lab results, the sensivity pattern can be very discouraging. Common ones like penicillinns (amoxyl) Cephalosporins (Ceftriaxone) etc, and only sensitive to floroquinolones and imipenem which are not cheap. (c) it becomes difficult to start requesting for drug like imipenem to teat common urinary tract infection

T2: very frequently, at least from looking at the laboratory results even without going to see the patients. It’s very common. (b) the gram negative enterobacterioceae such as E. Coli, klebsiella, with very high resistance profile from hospital sensitivity report, we have also seen gram positive cocci but not as much as the ones I mentioned before. (d) one of the peculiar things in this hospital is the way we have infection prevention control (IPC) unit and microbiology department. Our IPC is domiciled in community health, because they have a role but for surveillance it should be from microbiology department which should ideally be distributed to all units. We are not the ones in charge, IPC does that.

P1: We don’t have such

P2: We have a laboratory in this PHC. We also try to maintain records and documentations of tests conducted, but we do not have a dedicated report for antimicrobial resistance or surveillance.

P3: Here in Oredo, we do not have a laboratory, what we do is partner with the state hospital where we take samples to.

P4: there is no laboratory here, patients are referred to private laboratories

**What factors do you think are contributing to increasing emergence and spread of antimicrobial resistance? Please explain on how commonly each of them exist in your hospital?**

S1: A major factor I see is sales of antibiotics as OTC. We have strong resistance to cephalosporins and floroqunolones right now because almost everybody use antibiotics based on combinations given to them by patent medicine dealers and some pharmacies. Other situation where most or if not all antibiotics are given as POM will reduce resistance.

S2: empirical use of antibiotics and availability as at when due.

S3: one leading factor is poor infection control, another is overprescribing of antibiotics and patients themselves not completing dose of antibiotics.

T3: part of it is inappropriate use of antibiotics and poor infection control. A patient that has already been colonized with microorganism can easily spread it to others in the hospital, though the IPC committee which has been active since 2 years has been doing very well in this aspect. I am working closely with them to get baseline data that we can compare with to have evidence that the program is really impactful.

4. **What are your thoughts about Antimicrobial stewardship programs in Nigeria?**

**(B) What are your thoughts about the government’s involvement on AMS in the country with regards to the national action plan for antimicrobial resistance? (C) How will you describe the current use of antimicrobials in your facility in terms of appropriateness?**

T3: I know AMS is just coming up in many institutions in Nigeria. In our institution, we don’t have yet, we just set up a sub-committee from the DTC. With time after kick off, we can meet with management to have a stand-alone AMS committee. In most institutions in Nigeria, there is none.

If there’s an official document with the management, I am not aware (NAP). For now, it’s a voluntary thing a part of our professional responsibility and not as a mandate from management. (B) we are trying to gather data to show to management, so they can support it. It is worth spending money on.

T2: Nigeria as a whole, we are not really serious with AMS, we are not driving it as other nations are doing. (B) Government involvement if they are committed will have positive impact, in addition to policy makers. I am not aware of antimicrobial guideline, what I am aware of is guideline for use of drugs in hospitals i.e standard treatment guideline. I cant say its being in use.

T1: I buy the idea (B) it needs political/will power. If government participate, it will go a long way to help (C) average.

S2: it will help a lot, because if directive comes from above, everybody will be in line. If there’s a guideline all facilities can comply. For IPC, we are aware of little, little things they do. With regards to AMS, we have to put our feet down, and otherwise the resistance that will hit us as a nation, we will not survive it. You know if there is no guided use of antibiotics, we should have restricted use and stop exposing people to unnecessary use of antibiotics, so that they can naturally develop immunity to fight mild infections. It should not just be a policy but enforcing it. To enforce, there should be a kind of enlightenment so that everybody will be aware. This trickles down to healthcare workers. So there will be a team to let people know what antibiotics do to the system. In case one needs superior antibiotics, it would be that the patient is already exposed before. In developed countries, they are afraid talking of superbugs now even as they streamline their antibiotics use, we are not concerned with over-access. Some facilities should not have them at all like primary healthcare centers.

S3: with the advent of Covid, IPC programs got a boost. A lot of centres without IPC or inactive ones were either put in place or reactivated. I also think that with the proper policy, AMS can be put in place for health facilities. Some centres now have a drug policy unit like our centre has DTC.

**5.** **Does your hospital have a formal AMS program?**

**If yes, can you say in an ideal sense that it is functional? can you describe the structure and composition in terms of (i) formal policy(ii) Dedicated Team (iii) local antibiotic treatment guideline (iv) Tracking and reporting system (v) dedicated funding (vi) monitoring and evaluation.**

T3: No, but in the making

S1: None that I know of.

T2: we have IPC, but AMS is what management is on seriously building on DTC.

S3: for the AMS, we don’t have, but for the IP, I know theres a doctor that has come to talk to us on IPC. But I know theres IPC committee in the hospital.

T1: I know a committee was set up years back on IPC, but I don’t know if it is functional. For AMS, I don’t know either. A child was rushed in sometime ago and the attending doctor prescribed orelox (cefpodoxime), I queried it, but the doctor said he didn’t want to take chances. If orelox is prescribed for upper respiratory tract infections, what will be given for blood infections? Many mothers now, once their children runs temperature, you’ll see them rush to buy amoxyicillin or septrin. There has to be a reason for using antibiotics.

S2: No

P1: No we don’t

P2: That’s is new to me. (After Explanation), as for that, It’s not functional in this center

P3: I don’t know about that. (After Explanation) If clinicians are not passionate about antimicrobial stewardship, the clinical outcome for patient who are under their management would be poor because you have to be sure of the antibiotics most appropriate for a particular patient.

P4: I don’t really understand what it means. But if it has to do with effective use of antibiotics then it is a program that should be encouraged and taken seriously. (After Explanation), Okay, in that case, we do not have it here. Antimicrobial stewardship program would help us optimize the use of antibiotics and reduce the problem of resistance

**6. Are there target antibiotics that are either restricted in use or listed under antibiotic use surveillance in your institution? If yes can you mention them?**

T3: None right now. No one is monitoring that. Its what is prescribed by doctors that is supplied. That’s part of what the AMS will do. For example, there should be justification for prescribing Imipenem or meropenem.

T2: No

S3: None that I know of. The hospital has not made that known to us.

S2: Not really

S1: None that I know of. I haven’t encountered any that I have been told is restricted.

P1: (after explanation of antibiotic use restriction), We do not have restricted antibiotics, we use different class of antibiotics like amoxicillin, ciprofloxacin, erythromycin, gentamicin etc.

P2: The antibiotics we use are based on the essential drug list for primary healthcare.

P3: We have an essential drug list we try to adhere to. For certain antibiotics like Ofloxacin, the PHC head will have to consult the PHC coordinator before using, same procedure is applied in even some antibiotics may not be restricted.

P4: We have access to some antibiotics like cefuroxime, amoxicillin, metronidazole that we prescribe to patients. For cases that are beyond us, we refer to state hospital.

**Does your institution have antimicrobial consumption data at both patient level and facility level?**

S1: No

S2: No

S3: None in pharmacy, don’t know if the infectious disease department has.

T1: No

T2: no

T3: part of what we plan to work on, but none for now.

**7. What do you think are the barriers to AMS in your institution and the country at large?**

T3: management backing/support, without it one can’t go far on a voluntary basis unlike IPC which is active here, it has helped us, before the pandemic, handwashing and educational/awareness were already instituted here and was helpful during the pandemic. AMS is even more difficult because we are going to experience friction e.g autonomy in auditing prescriptions, without management backing it won’t work. (2) manpower- AMS is an intensive program, we are few with other professional duties, so there will be need for dedicated staff. (3) Interprofessional rivalry: e.g one of the things with bringing guideline for hospital use is if the hospital has an antibiogram. To do this effectively, we need very good working relationship with pharmacist, doctors, clinical microbiologists etc. in most institutions in Nigeria, there is this rivalry that may make it difficult to do seamlessly.

S3: in the institution, reporting is a problem, poor laboratory services like we talked about. Antibiotic resistance can be ascertained by laboratory investigation even up to malaria resistance. It involves testing. In the state, generally, there’s a dearth in manpower. Imagine no qualified staff to run those specialized laboratories, it can be difficult. There’s high level of under-staffing, management here has been supportive to our activities, so I think they will drive such program as AMS.

S2: institution, likely barrier is our professional rivalry, it’s for everybody to realize that we are in this workplace for the benefit of patients. Secondly, we need more staffs because we are already overstretched due to work overload. Management here is supportive. In Nigeria, financial inputs, because money is involved in running programs and implementing policies.

T2: one of it is that we have not really known the weight of the problem, there is no commitment from the leadership of the hospital. In Nigeria, we have this general attitude of not implementing policies. We already have the go ahead that institutions should start AMS, but we always end up with paper work

T1: bureaucratic bottle necks, professional/office politics, lack of understanding or awareness of the benefits it brings.

S1: in Nigeria, policies and implementation problems. In my institution, inadequate education of health professional and update on current use of antimicrobial guidelines is a big challenge.

**8.** **What can you say about how to mitigate these barriers?**

S3: leadership: if the leadership of each institution is committed that will be one way out. Then if the country is prepared to fund the program, we are good to go

T2: government can do the needful to employ more workers, more education on reporting antimicrobial resistance is important.

**9.** **What do you think are the facilitators of AMS programs in your institution that you can build on?**

T1: we already have a crop of professionals who are interested in this program, the necessary professionals are present in this hospital.

T2: the DTC has been inaugurated and can be built on.

S2: DTC, currently due to staff strength depletion, the committee is not meeting up to its responsibility. It’s a new committee. The pandemic made transfer of staff to the isolation center for Covid, that has affected its running.

S3: the DTC which is functional. It has already set up an AMS group which if not for Covid would have kick-started. Our present leadership is kind of interested in making it happen.

T3: DTC in the hospital, we have taken the initiative of grooming a pharmacist to go for training and inform the management through the DTC. The information technology unit in the hospital can be leveraged on. For example, the antibiogram I talked about can be generated from the infrastructure already on ground.

**10.** **Do you think your institution has the capacity to fully implement, be committed to and sustain AMS program?**

S2: yes

S3: yes it does

T1: yes, but it can be improved in terms of human capacity. For HIV, TB, Pneumonia, there are national guideline, but at local level, we don’t have.

T2: I think so. Its just the will power and interest . we have to prove to management the benefit of such program because a whole lot is involved. For example, IPC do a lot of things now compared to when they started, they are trained and retrained. It’s because management believes the unit is of benefit, that’s why they support it.

T3: I believe we can

**11.** **A key aspect of infection control is hand hygiene, what can you say about this practice among healthcare professionals in your institution?**

1. **Alcohol based handrub/water and soap always available at all hand washing basins? (ii) Enough hand washing facilities in your institution (iii) Routine education/ training of staffs**

T3: before the pandemic, hand hygiene was at a good level, during the pandemic, there was more improvement. Before, it was better than other centres I suppose, and ore people realized the benefits. Now, improvement will only be marginal.

T2: that has greatly improved with coronavirus infection which currently does not have a cure. So people are focused on prevention. Hand hygiene is improved in the hospital among other non-pharmacological measures. Thanks to the IPC team who always enlighten us on this. (b) alcohol hand rub was only available in few centre, but now it’s everywhere due to Covid. Hospitals management put water and soap on strategic locations. That’s one of the gains we receive in a third world country like ours.

S2: to an extent everybody is doing that during the pandemic. People are strict about it. Each unit has wash hand basin and water. Management has provided liquid soap too. But we can do better in a modern way, instead of bucket we should have running taps in work stations and toilets. Nowadays, you don’t even touch ta heads, they use sensors, same thing goes for soap, this helps control/prevent infection.

S3: hand hygiene, I think with the advent of Covid, a lot of us are on the alert, unlike before when we don’t care. But during lockdown and now, I think it is dying down. The IPC unit needs to revisit and create more awareness and motivation. (b) yes we do. The pharmacy department produces hand sanitizer for the hospital. When Covid just started, there were a lot of points with hand buckets for washing, I think those points have reduced now.

T1: IPC committee came to train us during Covid, sustaining is always the issue. Hand washing basins and water was everywhere then but now they are few.
