# Supplementary material for: Health care systems administrators perspectives on antimicrobial stewardship and infection prevention and control programs across three healthcare levels: a qualitative study
Source: Antimicrob Resist Infect Control. 2022 Dec 10;11:157. doi: 10.1186/s13756-022-01196-7 (PMC9739345; doi:10.1186/s13756-022-01196-7)
Supplement: Supplementary file 4 — Additional file4. D2: IPC Qualitative Transcription. [file 13756_2022_1196_MOESM4_ESM.docx]

**Infection Prevention and Control Program (IPC) Qualitative Interview Transcription**

1. **Please explain your clinical role and how it relates to your hospital’s overall infection control strategy?**

P1: public health physician, one unit in department is IPC, and head of unit, chairman of IPC committee in the hospital. Focal person for the orange network of National Center for Disease Control, for UBTH. The orange network is made of 34 facilities in country helping to ensure IPC practice is the high level of IPC practice in the country.

P2: I am the chief nursing officer IPC in UBTH. As a nurse, I advocate for proper hand washing. This involves using automatic soap dispenser with a proper antimicrobial soap. I also advocate using a hand dryer instead of using a towel as sharing a towel can lead to the transfer of microorganism.

S4: I am the focal person for the state, we receive reports on IPC from the state and private hospitals in the state, we go for monitoring every hospitals which has a focal person that has been trained on IPC, sometimes we go for supervision to ensure that they do what they are supposed to do. I am the director of nursing service in the ministry.

S5: I am not directly involved in IPC, but through my nurses in the 34 hospitals, although 32 are functional. I am the director of nursing services for the state’s hospital management board (HMB).

P1: IPC is usually coordinated by the head of the primary health care centers (PHCs). I am the coordinator in this PHCs in this local government area. The head of the PHC is in charge of supervising, monitoring and making sure that proper IPC practices are adhered to and reports to the coordinator of the local government. I as the coordinator report to the state coordinator of IPC.

P2: I am the Coordinator of all PHCs in Ikpoba-Oha local government area. It is my duty to ensure that proper infection control takes place in all centers here. I believe that infection control not only prevents infection and reduces spread, it also decreases reliance and use of anti-infective agents.

P3: I am the Oredo local government coordinator of PHCs.

P4: I am the Coordinator of PHCs in Ovia North East local government

1. **Do you think inappropriate use of antibiotics is a problem in Nigeria and in your institution? Which antibiotic use problems are more prevalent or common in this setting? How do you describe the pattern of this problem over time?**

T4: It leads to antimicrobial resistance (AMR). Which is a global public health threat among the top 10 by World Health Organization (WHO). It is a huge issue, we are getting to a stage where the resistance is advancing to multidrug resistance, simple infections usually treated with simple of single antimicrobial agents have now become resistant needing more expensive drugs. AMR is caused by antimicrobial misuse and overuses. In my setting, the microbiologist will be more in tune with that, we are going to do antimicrobial survey in this facility, but many infections are becoming resistant to common antibiotic particularly from wound swabs, implying that patients must have misused antibiotics prior to being admitted or in the case of healthcare associated infections (HCAIs), it can be resistant in out-patient too. Since there’s no baseline survey, we can’t really state that here although from the data seen elsewhere, it is increasing.

T5: Of course it is an issue in Nigeria and its healthcare facilities.

S4: it should be, these drugs are easily accessible everywhere in the country. I am not directly working in a healthcare institution, that is not to say that inappropriate antibiotic use does not exist there.

S5: I agree it is a major problem in our country, if nothing is done about it’s burden will increase in terms of resistance.

1. **How frequently do you encounter hospital acquired infection and resistant organisms within your clinical practice? Which types of pathogens/infections do you think have more resistance profile?**

T4: Yes we do, HCAIs. We have nurses who track such infections based on patient stay in the hospital and other criteria. The IPC repot tractks the pattern. It shows co-agulate negative, staphylococcus aureus, pseudomona aereuginosa, Klebsiella spp, providential spps, Enterobacter, citrobacter, alkaliginase,. The highest is coagulase negative staph aureus, and the lowest alkaliginase and citrobacter.

T5: The unit head has more information on that, but I can assure you that we do.

S4: we don’t have such data

S5: I can’t say for sure

1. **Do you usually have a regular surveillance reports of resistance and susceptibility pattern shared among healthcare workers**?

T4: yes, for resistance and sensitivity pattern, I don’t know if the microbiology do that, but monthly and quarterly reports are sent to management since they drive policy and are involved in decision making, also reports are sent to ward managers of affected wards on prevalence and pattern of HCAIs, so that if we are seeing an increasing patter, it shows they are doing something right or vice-versa.

S4: No

S5: No we don’t

1. **What are your thoughts about IPC programs in Nigeria in relation to the National Action Plan for Antimicrobial Resistance (NAPAMR)**

T4: In my opinion, the country has woken up in terms of IPC, the eye opener was Covid pandemic as well as infection transmission rate between healthcare workers and patients. With AMR, there is huge outcry in terms of resistance pattern for antihelminthics, antiviral, antibiotics even communicable diseases like TB. We are seeing a lot of MDR; policy direction should be in line with reducing infection in hospitals and AMR for communities.

**A lot of professionals have not heard of NAP/AMR, publicity is low, why is that so?**

Because of being in the orange network, a key advantage is that we are exposed quickly to national policies and guidelines especially from FMOH and NCDC, so we are abreast with what is happening at the national level. It is a 5-year proposal plan, 34 facilities are to do baseline AMR survey for the other facilities that can be used to form an informed decision and take action.

S4: we have IPC team, train them on environment and how to keep it clean.

S5: There’s no formal infection program in each institution as you have described, but the state government through the ministry of health encouraged constituted a committee, that is the IPC -Covid team as a response to Covid-19 control. It is this committee serves the state.

1. **Does your hospital have a formal IPC program, formal policy, team, guideline, funding, tracking and reporting, monitoring and evaluation?**

P1: yes to all. Oh yes. This is our policy document, its here with me like my bible, we have a reporting channel. We do health education for all staffs, e do staff orientation for new staffs, we know that IPC is a huge thing for any health facility, so that patient and healthcare worker leaves the facility worse than they came, so the minimum guideline should be followed, that is standard precaution.

S4: None that I can think of. I have trained IPC activities in almost all hospitals in Edo state. We have a focal person representing the state. They send things that they need, the logistic pillar provides these materials to them. We have a platform where we monitor their activities. We train pharmacists, Patent medicine dealers and recently even police. Funding is from the state government but not dedicated.

S5: I don’t think we have a formal IPC as described, but part of what Edo State IPC pillar is doing is to inform the various hospital and the public on prevention of nosocomial infection which is key to preventing spread of infection

Part of what Edo is doing is to inform the various hospital doing this and the public on prevention of nosocomial infection which is key to preventing spread of infection.

1. **What is the role of training in your activities, and how often?**

T4: it’s very important, last year alone, we had nothing less than 8 trainings, educating healthcare workers on hand hygiene and waste management, because we know that the hands of these healthcare workers are the most important vehicle for transmitting infections. We had to do a baseline survey of our hand hygiene facilities, Covid had a good side on hand hygiene, before now, we had soap and water, now, pharmacy department has been mandated to ensure hand sanitizers are produced and available in all wards and clinics. Pocket hand sanitizers was distributed to every health worker even to interns. We know people from the community come to our facilities like high risk areas such as special baby care unit. Our staffs go there and educate mothers and also distribute hand sanitizers to them. In addition, we now automated hand dryers and dispensing soaps in our wards.

T5: we train and retrain staffs and students. In clinical areas, we get feedback from healthcare workers on challenges they face, they tell us during our inspection routine.

S4: that’s the bedrock of IPC. Training helps to reinforce knowledge already acquired and also add new knowledge. It’s something that we do as part of the professional mandatory professional program for nurses. Some modules in the program include IPC. In a year we train up to 8 times. It is not solely on IPC, some modules train on IPC, but when you teach and nothing to back it up, like no materials it is like a waste.

S5: almost every month, we do training in various organizations and in different local government areas across the state. We just trained members of the police force. Once there’s a call on an outbreak, we call for training.

P1: we are all trained for it but I act as an overseer and coordinator of IPC activities in this PHC facility

P2: IPC has been taught in our health center, we have the posters and banners on how to keep infection out of the place all around

P3: we have undergone several trainings on IPC in the local government level and even at the state level. We have had at least two IPC training over the last eight months at local government level and state level each.

P4: actually, on IPC, all our staffs and health workers in the PHC have being trained, so they in the individual PHC know what to do

1. **(a) What can you say about your activities with regards to ensuring provision of adequate PPE for staffs and patients**

T4: we have a logistic unit that ensures we have all PPEs (face masks, hand gloves, head covers, foot covers, eye goggles and complete hazemat suit). In fact we had an indigenous production of some PPEs especially during the hit of Covid as there was tendency of worldwide shortage of these equipment, like I said before, Covid has its good side, because we started making things, cloth face masks for our admin personnel, long sleeve cover, aprons and face shield. We also made UV light equipment that uses UV light to disinfect some of our PPEs like face shields and eyewear. After soaking and washing, we disinfect using the UV machine. If we didn’t respond the way we did, our hospital would have recorded more healthcare associated infections than we did.

T5: we have a platform, if they lack any material, they forward and we send to them

S4: The IPC-Covid pillar is an arm of Covid committee. Part of what we do is ask hospital what materials they need so we can provide them. The Covid pillar has an IPC team focused on Covid. The committee then informs the government, some staffs in central hospital for example Director is a member of the committee. It is this IPC-Covid pillar that is usually called IPC team and liaises with all hospitals in the state.

P1: We receive PPEs from the state government through the COVID-Pillar. But the materials are not always enough.

P2: well we have challenges with resources in terms of equipment and welfare. Sometimes we run out of hand glove and other physical protective equipment (PPE) and before we are supplied it takes days so we need to buy with our money while awaiting the supply

P3: we are always short of hand gloves and face masks; we sometimes get these ourselves for fear of getting infected.

P4: On supply of IPC materials, I would give credit to the past local government area chairman, UNICEF and WHO, who has been diligent in providing constant supply of IPC materials.

1. **What can you say about your activities with regard to ensuring provision of adequate waste disposal to avoid spread of infections**

T4: if you go round our wards, you wee that we have color copded bin liners (red, tallow and black). Our waste is segregated at source. After that at strategic places in the hospital, we have Dino bins that are also color-coded, bin liners are transported and deposited into the dino bins, so that environmental health officers and waste managers can take the red and yellow bins to the incinerator, while the black coded bins goes for waste disposal. We have an incinerator in the hospital. In terms of waste management, I think we are doing well. we also have sharp boxes and ensure they are not over filled nor exhausted in the wards and clinics.

T5: wastes are segregated into infectious, highly infectious and non-infectious waste. For example, gloves used after attending to patients are placed in infectious (yellow-coded) bins before they are taken to the incinerator. Blood stained materials or wound dressings are placed on red bin liners as highly infectious waste. They are also incinerated, while black baskets are for wastes like water sachet, food containers or wrappings. Sharp boxes used to dispose of used injections, surgical blades and the likes are well disposed to.

S4: we have adequate incinerators for disposal of waste.

S5: We have central waste disposal. I think we still need training on segregation where the waste is generated, we just dump everything together which can be risky for those disposing it eventually, that is scavengers, then good sterilization is needed especially in rural areas where they boil their instruments, that will help

P1: We have safety boxes for disposal of injection needles and other sharp waste like broken ampoules. For waste that require incineration we use the incinerator at UBTH and sometimes the WHO facility. Most of the respondents in PHC facilities stated that they take wastes that require incineration to University of Benin Teaching Hospital or the World Health Organization waste disposal facility.

P2: Our waste are just medical waste, so we have safety boxes, covered waste bins for non-sharp and then the we drop sharp in the safety boxes. We don't have incinerators, so normally we gather the waste and send through the general waste collectors, then for medical waste that require incineration we send them to UBTH for incineration. When we do program such as intervention immunization, we are required to send the waste to WHO waste disposal facility

P3: We have a biohazard bag we use for collecting of waste generated in the laboratory. After usage, the bag is taken to the burn and bury site and burned immediately

P4: We ensure our environment is always clean, the cleaners actually do a good job on that. The waste accumulated are usually taken to incinerator for proper disposal.

**(c) What can you say about your activities in ensuring safe & clean environment, what has been put in place in this regard?**

T4: when we talk about clean environment, we are essentially looking at hand hygiene, toilets and the wards, and waste management. Our environment is regularly cleaned, I am sure when you go around the hospital, you will agree with me that it is clean. Now and during the hit of Covid, we use 0.5% sodium hypochloride solution to clean the floor, the walls and high touch areas such as door knobs and switches even the bedding and mattresses, and everything with same solution. We clean the wards and clinics at least twice daily. That’s management solution to promote quality care to our patients, we don’t want patients to leave worse than they came.

T5: we have cleaners who take care of the environment. They mop as at when due. Every Wednesday, we have inspections. Cleaners have been trained already including other healthcare professionals on IPC practice. Cleaners take the bulk because they take care of waste generated. The cleaners have enough materials to do their job. They have those thick hand gloves to protect themselves. They clean about 2-3 times daily. In out-patient setting, they have hand washing set up with soap dispenser like we have in antenatal clinic.

S4: all our nurses tend to maintain universal safety precaution. When they have any challenge, they communicate to us. The IPC-Covid pillar monitors activities such as waste disposal of refuse. If waste are accumulated, it is my responsibility to inform the state government who in turn ask those in charge to remove the waste. On a qauterly basis, they send report to us, if there is any challenge, we send the authorities involved.

1. **What are your thoughts about your preparedness in the event of an epidemic or case identification since we are in an era where such eventualities are becoming more frequent**

T4: I think that in preparing for an epidemic, the basic minimum for any facility or healthcare worker is to practice standard precaution, because standard precaution presupposes that every body that we come in contact with has a communicable infection until proven otherwise. So if we handle all patients that way and additional precautions if we think an infection maybe airborne or droplet nuclei associated, then we are on the good side. Management should live up to expectations by providing PPEs reminders and standard operating procedures to help healthcare workers, so that when they are with patients with suspected infection that can cause epidemic, they know what to do. We already have standard operating procedure for Covid, Monkey pus, and Lassa fever, these are well distributed also.

T5: standard precaution is the order of the day. Universal safety precaution is just for health worker to prevent a particular infection such as hepatitis or HIV, to protect himself. Standard precaution involves protecting self, patients and visitors from contracting infections both known and unknown. it is wider in application and involves hand hygiene, PPEs, the environment and safe available water.

S4: we pray there’s none. I believe universal safety precautions when maintained regularly can help. If we are able to tackle Lassa fever and Ebola, we can for any other epidemic. I tell the nurses not to allow PPE to get exhausted before requesting.

1. **What are the challenges you encounter to implement programs in your institution? And what do you think can be done to improve your activities**

T4: we have no issue with management, it is fully in support of our activities. Right now, we are planning for world’s hand hygiene day and we have their backing.

Our greatest challenge is behavioural change among healthcare workers, you know we always have new set of workers, entrenching the culture of standard precaution in healthcare workers need continuous medical education.

T5: our wards do not have enough waste disposal materials. Sometimes they end up using red or yellow bin liners for non-infectious wastes and vice-versa due to inadequate supplies. Staffs also complain about shortage of PPEs, for instance, a ward that is supposed to have a carton of glove for a week ends up having four packets, in the process of opening counting and sharing among the nurses, it leads to contamination, that in turn can cause nosocomial infections, thus prolonging patient stay and added bills. Same thing goes for face masks.

I think management should recognize that this new development is not good for IPC. I feel some patients are willing to even buy these PPEs or pay more to get better care. We are looking at speaking with management about it.

S4: inadequate transportation and lack of compliance. Some people don’t believe Covid or infection kills Africans. The focal person sometimes forget the training they receive.

We need dedicated vehicle from government for the program, because we will have to write each time we need to dispose waste.

S5: the major challenge is providing the things/materials needed. Especially waste disposal materials that is color-coded bins (red, yellow and black) which are used to segregate waste at the point of generation is lacking in many hospitals. We have learnt to improvise using black cellophane on basket, but still a big challenge. The government supply, but you know bottleneck in Nigeria government, supply is short, with the few supplies, there’s delay in receiving them after making advanced request. The government Is responsible for the supplies.

P1: The major challenge we face is transporting the waste to the incinerator. At times, it takes months for the dedicated vehicle to come for pick up. In terms of government involvement, the government is supportive. They give us support in training, almost every health staff have been trained on IPC either directly by the state or through step down trainings. They provide us the equipment and safety gadgets and also supply us with a lot of physical protective equipment (PPE) even if sometimes they run out. They carry out publicity to support us and these training is really helping in improving IPC practices in our institutions. However, I must mention that it is actually the government partners that are doing these things

P2: we are understaffed, in fact that is a challenge in most primary healthcare centers in the stste. Some PHCs are non-functional as a result of this*.* Government involvement in IPC is okay but it lacks adequate coordination. WHO and other government partners are also very helpful.

P3: We do not have enough materials for use. For instance, wastes are supposed to be separated, in a situation where there are no sharp boxes, one ends up placing all wastes together, that is dangerous. Another challenge we face is shortage of staff. Some centers do not have doctors, nurses or pharmacists. They rely on just a community health extension worker who does all medical activities, so infection control activities will be poor in such centers. The government is responsible for waste disposal especially that of sharp waste. They also supply us vaccines but we buy other IPC materials by ourselves

P4: The government is involved in IPC and they are doing their work. We receive IPC materials from them. The government agency for waste disposal comes here every day to carry the waste generated in the PHC. They also carry waste that requires incineration to the incinerator. If the government can provide a special vehicle for each PHC, that will be fine.

1. **What can you say about the strength of your IPC activities**

T4: it lies on the fact that we have management backing, dedicated IPC team staff and willing healthcare workers who are willing to accept the changes we propose, especially as the change are driven by data.

T5: before now, we wash our hand recently it is becoming a part of our life much better than. We no longer use tablet soap but liquid. Our waste segregation has improved unlike before when everything is placed together. Those in charge of carrying wastes are no longer scared of risk to their health.

S4: training and retraining, provision of PPEs, the government is doing well in these regards.

S5: it is obvious we are doing things right. If you hear the statistics of people dying or infected with Covid, ours in the state is low.

1. **How do you see the sustainability of your activities in years to come?**

T4: as long as the IPC committee is sustained in the facility which is over 20 years now, as long as we have an enabling environment for the IPC committee to work, it will go far and be sustained. Because it is one thing to have an IPC committee, it is another to have management backing and favorable environment for people to work. We are enjoying that now, I hope it continues.

S4: if we get someone that has the zeal and not concerned about payment, it is likely there will be sustainability.

S5: on a scale of 1-10, I will say 5. You know as government changes, things can change.

1. **Comparing hand hygiene now and in the past what do you have to say**

T4: we did a research to look at hand hygiene practice in 2014. Prevalence was less than 50%, this Covid era was more than 80%. I know it was borne out of fear, it wasn’t a culture, but I know that now, if we look at the prevalence, we may not be more than 80%, but we certainly are not less than 50%.

S4: compliance rate is high, a lot of people are conscious of their immediate surrounding compared to before. People now have hand sanitizers which they use. That’s one good thing Covid has done for us, to be conscious of our environment.

S5: on a scale of 1-10, I will say 9. A lot of people wash their hands more often now like mental illness. They wash as soon as they touch something. We have bucket taps in strategic places where taps are not flowing and soaps around the hospital which promote this habit.

P1: Ever since the outbreak of COVID 19, health workers have been duly sensitized about the importance of hand hygiene. It has ever since being a regular practice for us. The practice among us health workers has been okay, although not as it was in the heat of Covid.

P2: It has almost become a way of life for all health workers right now. Before we touch patients, we wash our hands and after touching patients we wash our hands. It's the pandemic that actually drove home this practice. It is not as if we accepted and practiced it willingly. In the past our attitude was like nonchalant. It was more practice during the early months of the pandemic

P3: We have been practicing hand hygiene perfectly though the COVID 19 pandemic really opened our eyes to the importance and value of hand hygiene. It is slightly decreasing now, but in the health center the standard and protocol is maintained

P4: we engage in proper waste disposal, hand washing, sterilization of instrument, maintenance of aseptic technique. You can see our bucket with a tap outside for maintenance of hand hygiene by consistent washing of hands with a running water and detergent

1. **What are you doing to encourage interdisciplinary collaboration**

T4:we have resident doctors, consultants and nurses who are strictly involved in IPC. That’s for the unit, we do weekly rounds on Wednesday across the hospital. The IPC committee is multidisciplinary, it is headed by a public health physician, we have clinical microbiologist, laboratory technologist, public health nurses, environmental health officers, from the four key department we have people involved. we Have youth corpers posted to the unit.

S4: in my IPC, we have pharmacists, nurses, doctors, laboratory scientists and volunteers. So it cuts across all profession. That is because we all need each other to work as a team.

S5: yes there is interdisciplinary collaboration in the Covid-pillar. It is made up of all health discipline.

1. **How is your team embracing the use of technology to advance its activities?**

T4: oh, wonderful. Some of our meetings are through zoom, reminders, letters, events are through whatsapp. We are also looking at other ways, for example, in our intensive care unit, we have extractor and purifier to purify the air around. We are installing that in the special baby care unit too. We are embracing technology full time in terms of health education, and the kind of gadgets we use.

T5: our programs are organized for training we use laptops and projectors.

S4: we do power point presentation for training and retraining.

S5: we have a platform where requests are submitted, made and sent. We hardly meet physically now, all our meetings are on zoom.
